# *In utero* Diagnosis of Long QT Syndrome: Challenges, Progress, and the Future

**DOI:** 10.4172/2376-127X.1000e125

**Published:** 2015-12-20

**Authors:** Suhong Yu, T Ronald Wakai

**Affiliations:** 1 Department of Radiation Oncology, University of Rochester, New York, USA; 2 Department of Medical Physics, University of Wisconsin-Madison, WI, USA

**Keywords:** Repolarization abnormalities, Long QT syndrome, Fetal magnetocardiography, T wave alternans, Torsade de Pointes, Fetus

## Introduction

Congenital long QT syndrome (LQTS) is an inherited ion channel disorder caused by gene mutations that encode for cardiac ion channels. The incidence of inherited LQTS in the general population is estimated to be 1:2000 (0.05%) [1]. LQTS manifests as prolonged corrected QT interval (QTc) on the electrocardiogram (ECG) and magnetocardiogram (MCG), and it is strongly associated with cardiomyopathies, channelopathies, and sudden death at all ages [2–5]. Sudden death results from susceptibility to Torsade de Pointes (TdP), a highly lethal ventricular tachycardia. Little is known about LQTS in the fetus, but an important recent study suggests that congenital LQTS may account for nearly 10% of unexplained fetal demise [6]. LQTS, however, is virtually unstudied in the fetus because the fetal ECG is difficult to record during pregnancy and repolarization is not detectable by ultrasound [7,8].

Over the last decade, advances in fetal magnetocardiography (MCG) have significantly enhanced our ability to diagnose fetal rhythm abnormalities, including LQTS. MCG is the magnetic analog of ECG. Unlike the fetal ECG, which is strongly attenuated by the electrical resistance of the fetal skin and vernix, the fetal MCG shows relatively high signal quality. The efficacy of fetal MCG is supported by a substantial literature, including several review articles [7,9]. It has been shown that in 15–20% of cases fetal MCG impacts the clinical management of the fetus with, or at risk of, arrhythmia, through greater diagnostic accuracy and by providing supportive evidence for fetal intervention, treatment, or timely delivery [10]. Fetal MCG has also been used to diagnose LQTS in utero and guide successful antiarrhythmic therapy [11–16].

## Challenges and Progress

Assessment of repolarization using MCG is considerably more difficult in the fetus than in the neonate or adult due to the lower amplitude of the signal and the presence of large interference, primarily from the maternal MCG. Signal processing techniques are required to remove the interference and facilitate detection of abnormalities such as QTc prolongation and T-wave alternans (TWA), i.e. beat-to-beat variation in the amplitude of shape of the T-wave [17–19]. TWA is a rare, but significant, rhythm pattern indicative of cardiac instability.

In a recent fetal MCG study, the electrophysiology of LQTS in utero was characterized for the first time in a sizeable cohort, consisting of 30 fetuses at risk of LQTS [20]. Heart rate, waveform intervals, T-wave morphology, initiation /termination patterns of TdP, and TWA were assessed. Fetal MCG demonstrated high diagnostic and prognostic value. Based on assessment of QTc interval (QTc> 490 ms), fetal MCG was able to identify the fetuses that tested positive for LQTS with high accuracy (89%). Low-for-gestational age heart rate (< 3%) was also associated with fetal LQTS. Some fetuses diagnosed with LQTS had only low fetal heart rate and no family history of LQTS at the time of referral. In several such cases, LQTS was subsequently found in 1stdegree relatives who underwent testing as a result of the fetal MCG diagnosis. The fetal MCG findings also showed high prognostic value. Subjects that had TdP as fetuses or newborns showed the longest values of QTc (>600ms). TdP was also associated with other rare findings, including 2nd-degree AV block, TWA, and QRS alternans. Lastly, definitive detection of TdP was critical for guidance of in utero therapy, consisting of administration of magnesium and lidocaine, which was highly effective at controlling or abolishing TdP.

## The Future

While the efficacy of fetal MCG has become more widely recognized, the high cost of the technology, which is based on superconducting sensors known as SQUID magnetometers, has limited its widespread use. This situation will likely change in the near future due to a recent breakthrough in atomic magnetometry, leading to the development of the so-called SERF (spin exchange relaxation free) magnetometer [21,22]. The SERF magnetometer is the first alternative device with sensitivity equal to or better than that of a SQUID magnetometer. Its main advantage, however, is low cost. The required optical components are inexpensive because they are already used in commercial products, e.g. DVD players; thus, atomic magnetometers can reduce the cost of fetal MCG detectors by nearly an order of magnitude. Promising results have already been published, and commercial systems based on atomic magnetometers will be realized in the near future [23].

Fetal MCG is an enabling technology for the in utero detection and management of LQTS. Due to the ability to effectively treat TdP in utero, these capabilities can be lifesaving. We foresee a promising future for in utero diagnosis of LQTS and other life-threatening fetal rhythm disorders.

## References

[1] Schwartz PJ, Stramba-Badiale M, Crotti L, Pedrazzini M, Besana A (2009). Prevalence of the congenital long-QT syndrome. Circulation.

[2] Schwartz PJ (1997). The long QT syndrome. Curr Probl Cardiol.

[3] Schwartz PJ, Stramba-Badiale M, Segantini A, Austoni P, Bosi G (1998). Prolongation of the QT interval and the sudden infant death syndrome. N Engl J Med.

[4] Narayan SM1 (2006). T-wave alternans and the susceptibility to ventricular arrhythmias. J Am Coll Cardiol.

[5] Korhonen P (2001). Repolarization abnormalities detected by magnetocardiography in patients with dilated cardiomyopathy and ventricular arrhythmias. Journal of Cardiovascular Electrophysiology.

[6] Crotti L, Tester DJ, White WM, Bartos DC, Insolia R (2013). Long QT syndrome-associated mutations in intrauterine fetal death. JAMA.

[7] Strasburger JF, Wakai RT (2010). Fetal cardiac arrhythmia detection and in utero therapy. Nat Rev Cardiol.

[8] Wacker-Gussmann A, Strasburger JF, Cuneo BF, Wakai RT4 (2014). Diagnosis and treatment of fetal arrhythmia. Am J Perinatol.

[9] Strasburger JF (2005). Prenatal diagnosis of fetal arrhythmias. Clin Perinatol.

[10] Bettina Cuneo F, Ronald Wakai JFS (2009). Magnetocardiography in the assessment of fetal arrhythmias. Expert Review of Obstetrics & Gynecology.

[11] Cuneo BF, Ovadia M, Strasburger JF, Zhao H, Petropulos T (2003). Prenatal diagnosis and in utero treatment of torsades de pointes associated with congenital long QT syndrome. Am J Cardiol.

[12] Simpson JM (2009). Fetal ventricular tachycardia secondary to long QT syndrome treated with maternal intravenous magnesium: case report and review of the literature. Ultrasound in Obstetrics & Gynecology.

[13] Horigome H, Iwashita H, Yoshinaga M, Shimizu W (2008). Magnetocardiographic demonstration of torsade de pointes in a fetus with congenital long QT syndrome. J Cardiovasc Electrophysiol.

[14] Hosono T, Kawamata K, Chiba Y, Kandori A, Tsukada K (2002). Prenatal diagnosis of long QT syndrome using magnetocardiography: a case report and review of the literature. Prenat Diagn.

[15] Menéndez T, Achenbach S, Beinder E, Hofbeck M, Schmid O (2000). Prenatal diagnosis of QT prolongation by magnetocardiography. Pacing Clin Electrophysiol.

[16] Schneider U, Haueisen J, Loeff M, Bondarenko N, Schleussner E (2005). Prenatal diagnosis of a long QT syndrome by fetal magnetocardiography in an unshielded bedside environment. Prenat Diagn.

[17] Yu SH, Wakai RT (2011). Maternal MCG Interference Cancellation Using Splined Independent Component Subtraction. IEEE Transactions on Biomedical Engineering.

[18] Yu SH, Van Veen BD, Wakai RT (2013). Detection of T-Wave Alternans in Fetal Magnetocardiography Using the Generalized Likelihood Ratio Test. IEEE Transactions on Biomedical Engineering.

[19] Yu SH, Van Veen BD, Wakai RT, Fetal QT (2015). Interval Estimation Using Sequential Hypothesis Testing.

[20] Cuneo BF, Strasburger JF, Yu S, Horigome H, Hosono T (2013). In utero diagnosis of long QT syndrome by magnetocardiography. Circulation.

[21] Kominis IK, Kornack TW, Allred JC, Romalis MV (2003). A subfemtotesla multichannel atomic magnetometer. Nature.

[22] Johnson C, Schwindt PDD, Weisend M (2010). Magnetoencephalography with a two-color pump-probe, fiber-coupled atomic magnetometer. Applied Physics Letters.

[23] Shah VK, Wakai RT (2013). A compact, high performance atomic magnetometer for biomedical applications. Phys Med Biol.

